# Full-length and near-full-length genomes of human rhinovirus A105 detected in patients with non-respiratory tract symptoms in Osaka, Japan

**DOI:** 10.1128/mra.00228-25

**Published:** 2025-07-31

**Authors:** Kazuma Okada, Yuki Hirai, Yumi Ushikai, Atsushi Kaida

**Affiliations:** 1 Division of Virology, Osaka Institute of Public Health 91397 , Osaka, Japan; 2 Laboratory of virus control, Center for Infectious Disease Education and research, Research Institute for Microbial Diseases, Osaka University 34822, Suita, Japan; Katholieke Universiteit Leuven, Leuven, Belgium

**Keywords:** human rhinovirus, non-respiratory tract symptoms, full-genome

## Abstract

One complete genome sequence and two nearly complete genome sequences of human rhinovirus A105, which were detected from patients with non-respiratory tract symptoms, were determined.

## ANNOUNCEMENT

Human rhinovirus (HRV) is a non-enveloped positive-sense RNA virus that belongs to the genus *Enterovirus* of the *Picornaviridae* family. It is divided into three species, *Enterovirus alpharhino*, *betarhino*, and *cerhino*, which contain more than 160 different genotypes (1). While HRV mostly causes the common cold and lower respiratory tract illnesses (2), it has also been detected in patients with non-respiratory tract symptoms (nRTS), including myocarditis, pericarditis, and encephalitis/encephalopathy (3–8). However, genome data of HRVs detected from patients with nRTS are limited. Here, we report full-length and near-full-length genomes of HRV-A105 detected in three patients with nRTS in 2015.

As HRV-A105 was detected only in patients with nRTS, HRV-A105-positive specimens were selected for genome sequencing. Those specimens were collected from hospitals in Osaka, Japan, as part of a passive surveillance program, which was part of a national surveillance program for viral infectious diseases in Japan based on the Infectious Disease Control Law (9). Those specimens included nasal secretions (patients N-18, N-23, and N-46) and fecal specimen (N-46). Patient ages were 11 years and 10 months, 7 years and 8 months, and 1 year and 9 months. N-18 presented with mononeuritis and glossopharyngeal nerve palsy, N-23 presented with acute myocarditis, and N-46 presented with paraplegia. Viral RNA in the specimens was extracted using QIAamp Viral RNA Mini Kit (Qiagen) and used for cDNA synthesis. Primers used in the synthesis were random hexamer, oligo(dt), and the original designed reverse transcription primer for 5′ RACE (Table 1).

**TABLE 1:** Primers for detecting and sequencing HRV-A105

Experiments	Primer name	Sequences (5′ −3′)	Amplified region (nt)^*a*^
Detecting	DK001	CAAGCACTTTGTTTCCC	161–550
	DK004	CACGGACACCCAAAGTAGT	
RT-PCR and sequencing	HRV49-80	GTACWCTRKTAYTMYGGTAMYYTTGTACGCC	49–1,217
	A_1219RP	GCTAACTGGTGTTCTGGTATCATAGCAACAA	
	A_960FP	GCCACTGCTATAGACAAACCATCTAGACCAG	958–1,949
	A_1954RP	CTAAAGCGTAAACTACCTGTCCAGTGAGTG	
	A_1542FP	GTCCCAATTACAATATCAATTAGCCCAATGTG	1,537–2,507
	A_2512RP	TCATCACGTGTTTGTGATGTCTGGACATAT	
	A_2099FP	TATGGTTGTGCCATGGGTTAGTGCTAG	2,094–3,353
	A_3358RP	GTTGCTTCAGTGCAATCACATGTTGGTAT	
	A_2980FP	CTAGAATTGTACTGACCAGCACACACATC	2,975–4,572
	A_4577RP	TGATGTGATTGTGGGTGGAGCTAACAT	
	A_4400FP	GGATGATATCATGCAAAATCCAAGTGGAGA	4,395–5,870
	A_5875RP	GCTTTTTCAAAATCAACTTCTAATCTAGGG	
	A_5577FP	CCAACAAAATCAGGGTATTTGGTGGGAT	5,572–7,020
	A_7025RP	AGCGCCAACACTCGTATCCGCTTCTCAAA	
5′ UTR sequencing with SMARTer RACE 5′/3′ Kit (TaKaRa)	gsp-RT	GAAACACGGACACCCAAAGTAGTTGGTCCC	
	GSPv2-smarter	GATTACGCCAAGCTTAGCTGCAGGGTTAAGGTTAGCCACATTC	
	5utr-seq	CAGCCACGCAGGCTAGAACA	
3′ UTR sequencing with 3′-Full RACE Core Set (TaKaRa)	A_6729Fseq	GAGGAGATACATGAATCCATTCG	

^*a*^ Based on the complete genome sequence of A105 (accession number LC857137).

We detected the HRV genomes and investigated their genotypes using an approximately 390 bp region in the 5′ UTR amplified with the primers DK001 and DK004 (Table 1) (10, 11). These PCR products were Sanger sequenced and classified as A105 based on phylogenetic analysis (11). Other primers were designed based on genome sequences of A105 and A57, which are closely related, to determine the whole genome sequence of A105 detected in this study. HRV genomes, except the 5′ and 3′ end regions, were amplified by PCR using KOD-One (TOYOBO) with the primer pairs (Table 1). Seven overlapping PCR products were generated per genome with amplicon lengths ranging from 970 to 1,475 bp. Each PCR product was Sanger sequenced bidirectionally. The 5′ and 3′ UTRs were sequenced using the SMARTer RACE 5′/3′ kit and 3′-Full RACE Core Set (TaKaRa). Assembly used overlapping regions of 200–500 bp using DNADynamo v1.63 (BlueTractorSoftware).

**Fig 1:**
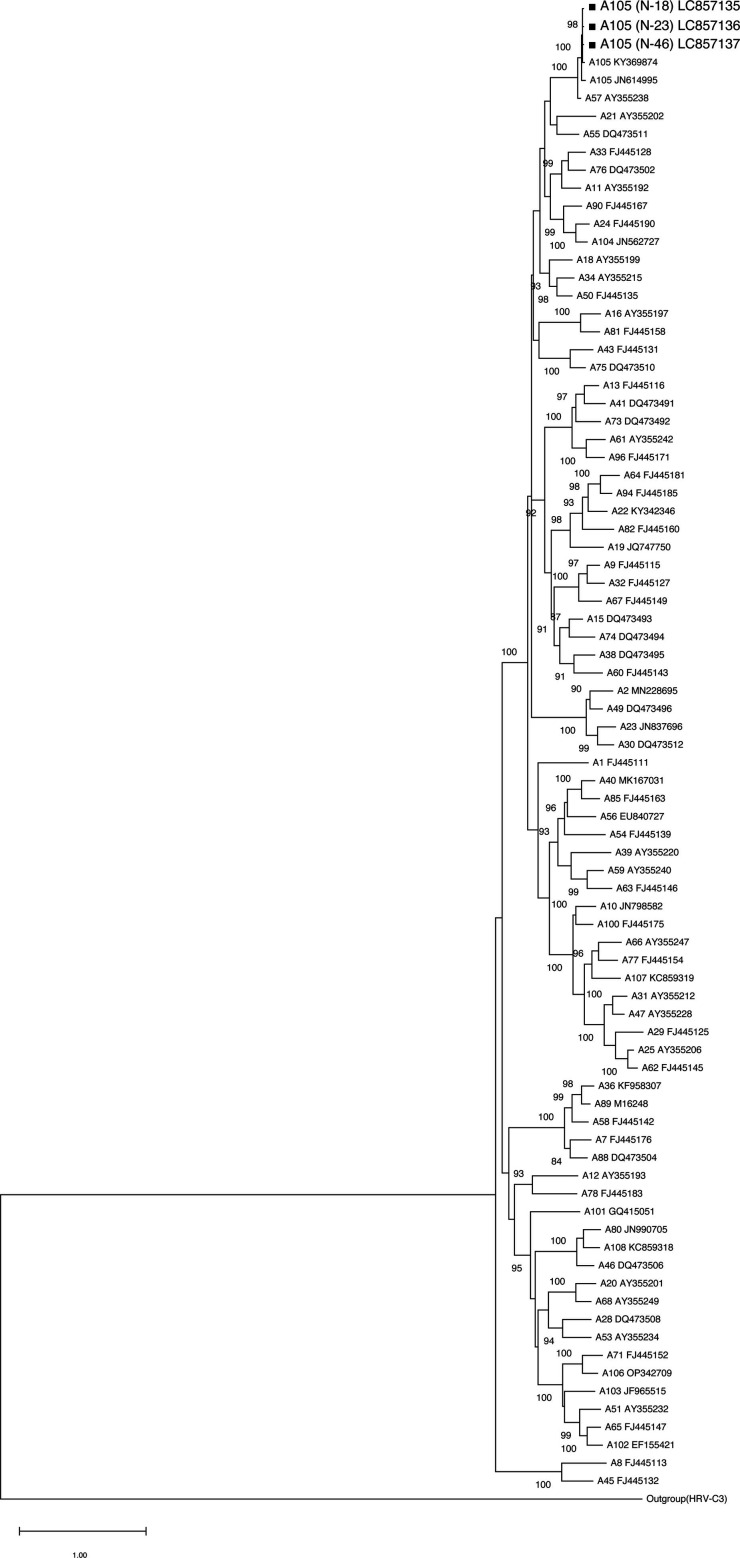
Phylogenetic tree analysis of VP1 sequences of HRV-A and HRVs detected in patients with nRTS in 2015. Maximum likelihood phylogram of the gene of the VP1 region (843–1,008 nt) of HRV-A strains identified in this study (black squares) and reference strains. Based on Bayesian information criteria, the general time reversible model was used to construct the phylogram. Numbers at the nodes indicate the bootstrap support values, which are expressed as a percentage of 100 replicates (values <80% are omitted). Each strain detected in this study is identified by patient ID. Other HRV-A strains are identified with accession numbers shown in parentheses.

The complete genome derived from the nasal sample of N-46 was 7,129 nt long and had a GC content of 38%. The nearly complete genomes derived from N-18 and N-23 were 7,058 and 7,063 nt long, respectively, and both had a GC content of 38%. The three HRVs were classified into the A105 genotype cluster by phylogenetic analysis based on VP1 nucleotide sequences (Fig. 1). The *p*-distance values compared to A105 reference strain SC9723 (KY369874) were 0.017 (N-18), 0.016 (N-23), and 0.019 (N-46), confirming all derived genomes as A105 genotypes (12, 13). While causal evidence linking HRV to nRTS remains limited, this study may contribute to understanding the pathogenesis of HRV.

## Data Availability

The viral genome sequences of HRV-A105 detected in this study have been deposited in the DDBJ under the accession numbers LC857135 (N-18), LC857136 (N-23), and LC857137 (N-46).

## References

[1] Esneau C, Duff AC, Bartlett NW. 2022. Understanding rhinovirus circulation and impact on illness. Viruses 14:141. doi:10.3390/v14010141 35062345 PMC8778310

[2] To KKW, Yip CCY, Yuen KY. 2017. Rhinovirus - From bench to bedside. J Formos Med Assoc 116:496–504. doi:10.1016/j.jfma.2017.04.009 28495415

[3] Hazama K, Shiihara T, Tsukagoshi H, Matsushige T, Dowa Y, Watanabe M. 2019. Rhinovirus-associated acute encephalitis/encephalopathy and cerebellitis. Brain Dev 41:551–554. doi:10.1016/j.braindev.2019.02.014 30850156

[4] Han S, Liu J, Feng Z, Mao Y, Gao H, Xie Z, Qian S, Xu L. 2024. Fulminant myocarditis associated with human rhinovirus A66 infection: a case report. Front Pediatr 12:1480724. doi:10.3389/fped.2024.1480724 39529970 PMC11551029

[5] Liu J, Zhao H, Feng Z, Liu Y, Feng Q, Qian S, Xu L, Gao H, Xie Z. 2022. A severe case of human rhinovirus A45 with central nervous system involvement and viral sepsis. Virol J 19:72. doi:10.1186/s12985-022-01799-x 35459180 PMC9034649

[6] Pelkonen T, Roine I, Anjos E, Kaijalainen S, Roivainen M, Peltola H, Pitkäranta A. 2012. Picornaviruses in cerebrospinal fluid of children with meningitis in Luanda, Angola. J Med Virol 84:1080–1083. doi:10.1002/jmv.23304 22585725

[7] Soma N, Aizawa Y, Matsunaga M, Saitoh A. 2021. Clinically mild encephalitis/encephalopathy with a reversible splenial lesion associated with rhinovirus. Pediatr Infect Dis J 40:e122–e125. doi:10.1097/INF.0000000000002995 33464018

[8] Tapparel C, L’Huillier AG, Rougemont A-L, Beghetti M, Barazzone-Argiroffo C, Kaiser L. 2009. Pneumonia and pericarditis in a child with HRV-C infection: a case report. J Clin Virol 45:157–160. doi:10.1016/j.jcv.2009.03.014 19427260 PMC7108322

[9] Kaida A, Kubo H, Takakura K, Sekiguchi J, Yamamoto SP, Kohdera U, Togawa M, Amo K, Shiomi M, Ohyama M, Goto K, Hase A, Kageyama T, Iritani N. 2014. Associations between CO-detected respiratory viruses in children with acute respiratory infections. Jpn J Infect Dis 67:469–475. doi:10.7883/yoken.67.469 25410563

[10] Kiang D, Yagi S, Kantardjieff KA, Kim EJ, Louie JK, Schnurr DP. 2007. Molecular characterization of a variant rhinovirus from an outbreak associated with uncommonly high mortality. J Clin Virol 38:227–237. doi:10.1016/j.jcv.2006.12.016 17276135

[11] Kiang D, Kalra I, Yagi S, Louie JK, Boushey H, Boothby J, Schnurr DP. 2008. Assay for 5’ noncoding region analysis of all human rhinovirus prototype strains. J Clin Microbiol 46:3736–3745. doi:10.1128/JCM.00674-08 18753359 PMC2576622

[12] Simmonds P, Gorbalenya AE, Harvala H, Hovi T, Knowles NJ, Lindberg AM, Oberste MS, Palmenberg AC, Reuter G, Skern T, Tapparel C, Wolthers KC, Woo PCY, Zell R. 2020. Recommendations for the nomenclature of enteroviruses and rhinoviruses. Arch Virol 165:793–797. doi:10.1007/s00705-019-04520-6 31980941 PMC7024059

[13] McIntyre CL, Knowles NJ, Simmonds P. 2013. Proposals for the classification of human rhinovirus species A, B and C into genotypically assigned types. J Gen Virol 94:1791–1806. doi:10.1099/vir.0.053686-0 23677786 PMC3749525

